# A Call for Incorporating Social Research in the Global Struggle against Snakebite

**DOI:** 10.1371/journal.pntd.0003960

**Published:** 2015-09-17

**Authors:** José María Gutiérrez, Thierry Burnouf, Robert A. Harrison, Juan J. Calvete, Nicholas Brown, Simon D. Jensen, David A. Warrell, David J. Williams, Global Snakebite Initiative

**Affiliations:** 1 Instituto Clodomiro Picado, Facultad de Microbiología, Universidad de Costa Rica, San José, Costa Rica; 2 Graduate Institute of Biomedical Materials and Tissue Engineering, Taipei Medical University, Taipei, Taiwan; 3 Alistair Reid Venom Research Unit, Liverpool School of Tropical Medicine, Liverpool, United Kingdom; 4 Instituto de Biomedicina de Valencia, Consejo Superior de Investigaciones Científicas (CSIC), Valencia, Spain; 5 Australian Venom Research Unit, Department of Pharmacology & Therapeutics, University of Melbourne, Parkville, Victoria, Australia; 6 Charles Campbell Toxinology Centre, School of Medicine & Health Sciences, University of Papua New Guinea, Port Moresby, Papua New Guinea; 7 Nuffield Department of Clinical Medicine, John Radcliffe Hospital, University of Oxford, Oxford, United Kingdom; University of Kelaniya, SRI LANKA

In Africa, Asia, Latin America, and parts of Oceania, envenoming after snakebite is a serious public health problem [1]. Conservative data suggest that between 1.2 and 5.5 million people suffer snakebites every year, resulting in 25,000 to 125,000 deaths and leaving approximately 400,000 victims with permanent sequelae [2,3]. Despite its significant impact on human health, this disease remains largely neglected by national and international health authorities, funding agencies, pharmaceutical companies, patients’ organizations, and health advocacy groups [1,2].

Most initiatives aiming to study snakes, snake venoms, and snakebite envenoming and its treatment approach the problem from a biomedical and technological perspective. Notwithstanding the substantial scientific and clinical legacy generated through this view, significant gaps remain in our understanding of other highly relevant aspects of this problem and its solutions. The emerging field of global health has brought about a more holistic approach to health issues by incorporating a “biosocial approach” to the understanding of diseases and the circumstances behind their occurrence [4]. The centrepiece of this approach is the integration of biomedical aspects—including etiology, pathophysiology, diagnosis, and therapy—with the analysis of the social, economic, psychological, cultural, and political contexts in which diseases occur. Snakebite envenoming is predominantly a disease of the poor [5], with the highest incidence and severity seen in regions facing complex and interrelated social and economic problems. Understanding how this interplay of variables influences both the circumstances leading to snakebite injury and its consequences is crucial to developing successful strategies to mitigate the problem. A comprehensive multidisciplinary approach incorporating social research into the study of snakebite envenoming is needed. Hereby, we aim to increase awareness of the following areas where reinvigorated social research would be highly beneficial.

## Dimensioning the Magnitude and Social Implications of the Snakebite Problem

A key issue undermining advocacy efforts to measure the impact of snakebite envenoming worldwide is the poor level of information on incidence, mortality, sequelae, and social suffering associated with this disease. Most studies are based on hospital statistics that greatly underestimate the burden. Well-designed, adequately powered, community-based surveys of snakebite incidence and mortality are required to provide reliable data (see [6,7], for example).

One key morbidity indicator, the Disability Adjusted Life Year (DALY), is rarely used to appreciate the impact of snakebite envenoming. We stress the need to evaluate the degree of broader “social suffering,” i.e., the effects on people with personal and economic links to the envenomed person. Gathering this information will be possible by using community surveys and household interviews to collect data on the circumstances of the bites, whether the victim attended health facilities, the extent and type of attention provided by local traditional healers, and the consequences and sequelae of envenoming, including physical, psychological, and economical aspects. We recommend that these surveys also identify socio-ethnic, occupational, biogeographic, and behavioural factors that remain undetected in conventional hospital-based or national-level public health data. These efforts should also shed light on the incidence of post-envenoming disability, both temporary and permanent. Few studies highlight the large impact of physical, psychological, and economic sequelae that result from snakebites [8,9].

## Identifying Barriers to Antivenom Access

Availability and accessibility of antivenoms is limited in sub-Saharan Africa, Asia, and, to a lesser extent, Latin America [2]. Such shortages lead to a vicious cycle whereby poor supply results in higher prices and lower confidence in the public health sector’s ability to provide effective and safe antivenoms [10,11]. We stress that a thorough understanding of the mechanisms of pharmaceutical supply, distribution, and affordability in regions with limited access to antivenom is crucial for devising improved accessibility strategies. Concomitantly, there is a need for economic analysis of both the sustainability of antivenom supply systems and the modelling of new economic strategies for financing production, distribution, and supply through to the end users. Cost-effectiveness studies of antivenom treatment should also be promoted [12]. Comparisons of approaches used for vaccines and other biologicals may provide useful insights. We trust that renewed research efforts on these topics will help in designing novel strategies for improving antivenom availability and accessibility.

## Exploring the Access to Health Services

Snakebite envenomings are medical emergencies that require prompt medical attention. Hence, a key issue for reducing their biosocial impact is rapid access to effective healthcare. In many snakebite-affected countries, an envenomed victim may need to walk (or be carried) for many kilometres to reach a primary health post. Thus, studies of the circumstances that delay the access of people bitten by a snake to health centres are of great value. Moreover, having antivenom in stock is not the complete answer. Rural health facilities also require the proper storage infrastructure, which encompasses cold-chain procedures and equipment and access to the other medicines, equipment, and consumables that are necessary to administer antivenom. Beyond initiating treatment, reliable communications infrastructures, transport routes, and ways to ensure the safe transfer of envenomed patients to advanced facilities are all needed [13]. We urge research on the organization of public health services and infrastructure in regions of high incidence of snakebites, as well as on issues associated with medical training and antivenom supply and use. Innovative schemes are needed and they require field studies, such as the centralized “hub-and-spoke” strategy proposed for Nigeria, which not only broadens antivenom access but also implements quality assurance, standardization, and training of health staff [14]. Likewise, a “diagonal” approach in primary health care should be explored, in which the prevention and treatment of envenomings parallel the general strengthening of public health systems.

## Improving the Training of Health Staff: How to Organize Teaching Activities Tailored to Local Needs

The skills of personnel at all levels of the health system are critical determinants of effective snakebite envenoming treatment. While much of this education needs to be integrated into the curricula of medical and nursing schools, it should also be included in continuing medical education programs, especially in areas where envenoming is common. Training programs should be designed around robust performance indicators that can be regularly evaluated to assess teaching outcomes, and evaluate the currency and appropriateness of their content. To this end, the preparation of national or regional guidelines for the diagnosis and treatment of snakebite envenomings is of utmost relevance. The design of training curricula should be guided by baseline research aimed at determining the current level of staff knowledge in different roles and settings. Information and communication technologies open the door to much wider dissemination of standard protocols; the selection of the particular technologies implementable in each local setting should be supported by a knowledge-based approach strengthened by renewed research initiatives.

## Understanding the Impact of the Local Context: The Perception of Snakebite in Different Cultural Settings

One of the great failures of “vertical,” top–down interventions in public health issues is the lack of understanding of the way local communities perceive health problems. Consequently, snakebite prevention and intervention programs at the community level should include in-depth analyses of the cultural characteristics of the communities, the way snakes and snakebites are perceived, the cultural background of local healers, and the perception of state-based, westernized medical services. The reasons why people bitten by snakes do not attend local facilities should be thoroughly investigated. In this context, ethno-anthropological research is a priority and should be readily implemented in regions of high snakebite incidence.

## Designing Strategies to Improve Prevention and Early Management: How Do Communities Organize Help for Snakebite Victims?

The two most critical aspects of any comprehensive snakebite management strategy linked to local settings and community organizations are prevention and early management of cases. Public campaigns for snakebite prevention require a detailed knowledge of the cultural features of rural communities and their organization and leadership through anthropological research. The use of local languages and dialects, and the involvement of the local population in the community programs, should be incorporated at early stages in prevention efforts. In general, the active engagement of communities in the development of health intervention programs greatly increases the likelihood of success [15].

## Final Remarks

We wish to stress that the approaches to confront snakebite envenoming as a public health problem must go beyond the biomedical and technological paradigms and should encompass socio-ethnic, socio-economic, and anthropological perspectives. The integration of natural and social sciences in the study of snakebite envenoming, in association with community-based, national, and international advocacy efforts, will certainly bring a fresh and renewed perspective towards understanding and reducing the dramatic burden of this complex and neglected health problem.
